# The dexter robotic system in gynecological surgery: preliminary clinical data from routine use

**DOI:** 10.1007/s11701-026-03338-0

**Published:** 2026-03-27

**Authors:** Huy Duc Le, Zino Ruchay, Julian Pape, Anna-Christina Rambow, Veronika Günther, Pernilla Virginia Conrad, Julius Pochhammer, Nicolai Maass, Ibrahim Alkatout

**Affiliations:** 1 Department of Gynecology and Obstetrics, University Hospital of Schleswig-Holstein, 24105 Kiel, Germany; 2 Department of General, Visceral, Thoracic, Transplantation and Pediatric Surgery, University Hospital of Schleswig-Holstein, Kiel, Germany

**Keywords:** Dexter, Robotic surgery, Gynecology, Hysterectomy, Operative time

## Abstract

The Dexter robotic surgery system is a novel approach combining conventional laparoscopic and robotic surgery through an on-demand hybrid concept. Following its clinical approval in 2022, it has been used in a variety of surgical fields, but data on routine gynecological application remain limited. This study reports the first consecutive case series evaluating technical feasibility and early outcomes of the Dexter system in routine gynecological surgery. This retrospective consecutive case series included all gynecological procedures performed with the Dexter system at a single academic center between November 2022 and March 2024. Primary outcome was operative time (skin-to-skin). A total of 47 patients underwent nine different types of gynecological procedures. Benign hysterectomies were the most common (*n* = 28/47, 59.6%). Mean operative times varied between procedures, with the longest duration observed for hysterectomy combined with cervicosacropexy (161.0 min ± 26.0 min), followed by total hysterectomy (139.8 min ± 33.1 min) and myomectomy (131.0 ± 42.7 min). Blood loss was < 50 ml in all cases. No intraoperative complications were observed and no conversions to laparotomy or conventional laparoscopy occurred. This study demonstrates the technical feasibility of the Dexter robotic system for routine minimally invasive procedures in gynecological procedures. The on-demand hybrid concept was successfully integrated into clinical workflow. These findings provide foundational data for future comparative studies evaluating the Dexter system in gynecological surgery.

## Introduction

The advent of laparoscopy signified a pivotal paradigm shift in the domain of surgical innovation, profoundly influencing and redefining the landscape of surgical methodologies. Notably, the field of gynecology played a crucial role, as gynecologists were among the pioneers in the early days of minimally invasive surgery [1]. In recent years, the use of robot-assisted laparoscopic surgery has undergone a significant evolution in this field and has enhanced benefits for both surgeons and patients. This has led to its widespread application in gynecological surgery for both benign and malignant conditions [2, 3]. Due to the successful establishment of robotic surgery, more and more robotic surgery platforms are being developed, accompanied by the ongoing refinement of existing platforms [4]. 

**Fig. 1:**
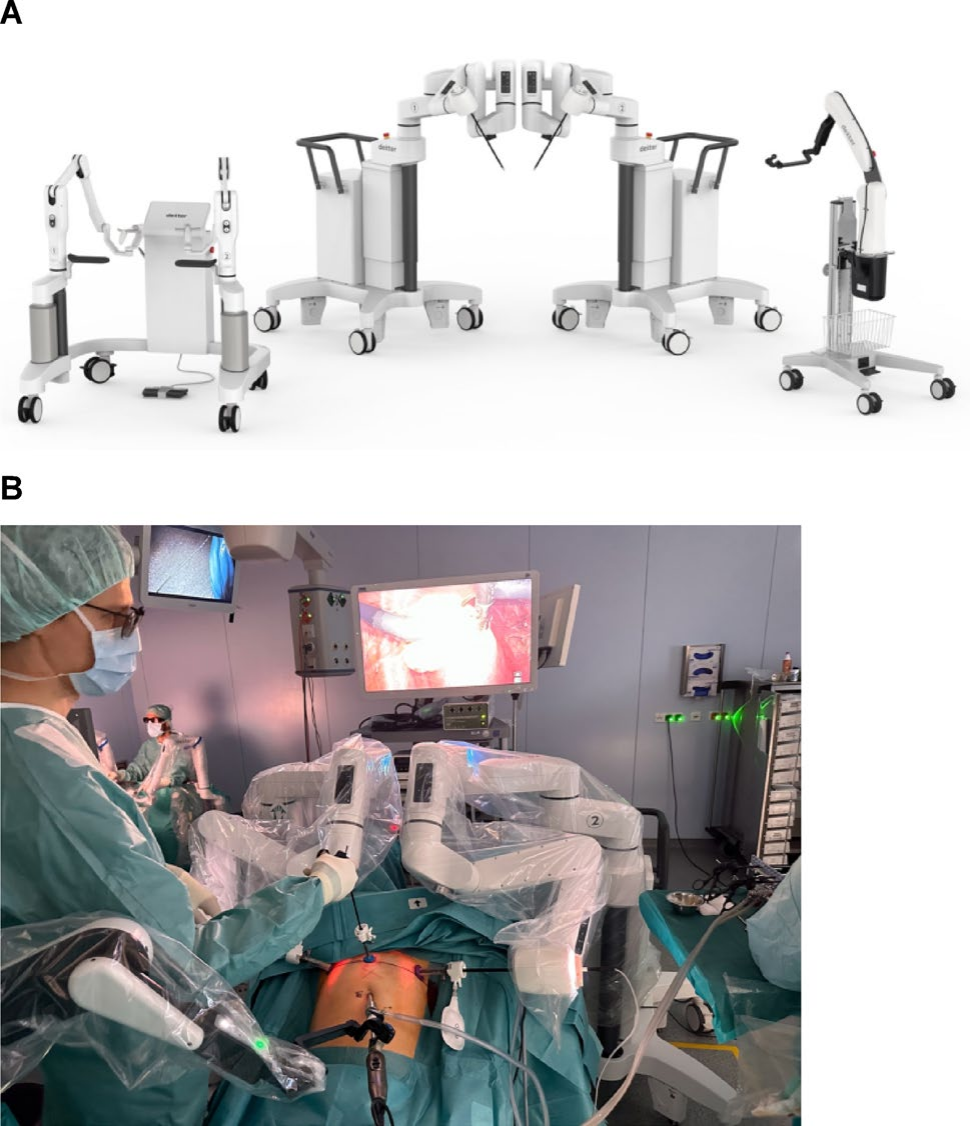
The Dexter robotic system consists of two patient carts, an endoscope cart and a surgeon’s console, allowing a seamless switch between the conventional laparoscopic and the robotic surgical approach. ** A)** Structure of the Dexter robotic system [5] **B)** The system in sterile use during an operation. The surgical approach is similar to that of conventional laparoscopy

The Dexter robotic system (Distalmotion SA, Epalinges, Switzerland) is a recently introduced robotic surgery platform that offers an on-demand concept through an innovative approach. It is an open, mobile and modular platform that employs the trocar placement of conventional laparoscopic surgery. Unlike traditional robotic surgery platforms where the surgeon operates from a non-sterile remote console, the Dexter robotic system features a sterile console. This unique configuration allows the surgeon to seamlessly transition between manual laparoscopic and robotic operation without breaking sterility or requiring re-draping. The system comprises two carts, each equipped with a robotic working arm, an additional robotic endoscope arm, and a surgeon’s console. This modular design enables selective activation of robotic assistance for specific surgical steps where precision and ergonomics are most beneficial, while maintaining the option for direct manual intervention when tactile feedback or efficiency is preferred. These features potentially offer advantages in workflow flexibility, cost-efficiency and surgical training compared to integrated robotic platforms that require complete robotic execution once docked. [Figure 1] [5, 6] A systematic evaluation of the Dexter robotic system in clinical practice is necessary to determine the efficacy of its unique hybrid approach, which combines conventional laparoscopic and robotic capabilities for each surgical field. The clinical use and potential of the Dexter robotic system have been described recently, particularly in the context of visceral surgery and urology, implicating a seamless integration into the existing workflows while maintaining the benefits of robotic surgery and thus being feasible for procedures in both fields [7–9]. However, comprehensive data regarding its application in gynecological surgery remain scarce, as existing studies have primarily focused on hysterectomies rather than exploring its potential across a wider range of procedures [10–12].

This study presents the first consecutive case series of the Dexter robotic system in routine gynecological surgery. The objectives were to: demonstrate technical feasibility across different procedure types, report operative times as benchmarks for future studies, assess the early safety profile and evaluate integration of the on-demand hybrid concept into gynecological workflow.

## Materials and methods

### Ethics approval and participation consent

The study was carried out in accordance with the Helsinki Declaration of 1964 and its later amendments, as well as the ethical standards of the institutional and national committee on human experimentation. The study was approved by the institutional ethics committee of the University Hospital of Schleswig-Holstein in Kiel, Germany (reference number: D 535/24).

### Study design and setting

This retrospective observational study included all consecutive patients who underwent gynecological surgery with the Dexter robotic surgery system (Distalmotion SA, Epalinges, Switzerland) at the Department of Gynecology and Obstetrics, University Hospital of Schleswig-Holstein, Campus Kiel, Germany between November 2022 and March 2024. The study period represents our initial implementation phase of the Dexter platform. No patients were excluded from analysis. After patients had been included in the study, their data were pseudonymized.

### Patient selection and case distribution

All gynecological procedures considered suitable for minimally invasive approach were eligible for Dexter-assisted surgery. During the initial implementation phase, case selection favored patients with ASA status I-II to establish baseline experience. No specific exclusion criteria based on BMI, prior surgery or uterine size were applied. Oncological cases requiring lymphadenectomy were not performed during this initial series.

### Surgical team and training

All surgical procedures were performed by two board-certified surgical gynecologists, both with > 15 years of laparoscopic surgery experience and > 50 prior robotic cases with the DaVinci system (Intuitive Surgical, Sunnyvale, USA). Prior to the use of the Dexter robotic system, both gynecologists underwent a training program by the manufacturer Distalmotion and subsequently acquired the manufacturer’s certification. The program included an online theoretical course, extensive simulator training with different surgical exercises and a combination of dry lab and wet lab hands-on sessions, ensuring the surgeons were well-prepared before transitioning to live surgery. A manufacturer’s clinical specialist was present during all procedures to provide technical support if needed. The specialist did not participate in surgical decision-making or data analysis. The surgeons operate from a sterile console positioned within the sterile field, allowing direct table access without breaking sterility.

Surgical technique followed standard principles for each procedure type. The hybrid functionality was utilized pragmatically based on intraoperative need, such as manual specimen retrieval, placement of additional ports or specific dissection steps requiring direct tactile feedback. The decision to use robotic versus manual laparoscopic technique for individual surgical steps was at the surgeon’s discretion.

### Data collection and outcome measures

Patient demographics, surgical indications, comorbidities and prior surgical history were extracted from electronic medical records. All patients provided written informed consent for the use of the Dexter robotic system and for data use for research purposes.

The primary outcome was operative time, defined as skin-to-skin from first incision to final skin closure, including all steps: port placement, docking, console operating time, undocking and wound closure. Times were recorded from standardized operation and anesthesia protocols. Secondary outcomes included intraoperative complications (defined as any unintended event requiring intervention), conversion to conventional laparoscopy or laparotomy, estimated blood loss in milliliters, blood transfusion requirement (intra- or postoperative), uterine weight for hysterectomies acquired from pathology records, length of hospital stay, 30-day postoperative complications according to the Clavien-Dindo classification and 30-day readmission or reoperation rates.

Regarding device-specific metrics, docking time was not separately recorded in operation or anesthesia protocols and is therefore included in total operative time. Based on manufacturers specification and surgeon estimation, typical docking time for the Dexter robotic system is approximately 5–8 min.

### Statistical analysis

Continuous variables are presented as mean ± standard deviation (SD) for normally distributed data or median with interquartile range (IQR) for non-normally distributed data. Categorical variables are reported as frequencies and percentages. For comparison of operative times between procedure types, the Mann-Whitney-U test was used for two-group comparisons. Statistical significance was set at *p* < 0.05.

Missing data were documented and reported. Complete operative time data were available for all 47 cases. Statistical analysis was performed using GraphPad Prism 9.0 (GraphPad Software, San Diego, USA) and Microsoft Excel 2019 (Microsoft Corporation, Redmond, USA).

### Data availability

The European Union General Data Protection Regulation law regulates and restricts public sharing of the underlying clinical data. However, aggregated anonymized data can be disclosed upon reasonable request.

## Results

From November 2022 to March 2024, 47 consecutive patients underwent gynecological surgery with the Dexter robotic system. Patient characteristics are summarized in Table 1. The median age was 41 years (IQR 36.5–48 years, range 24–69 years) with a median BMI of 24.4 kg/m^2^ (IQR 21.9–28.4, range 19.6–39.2 kg/m^2^). All patients were classified as ASA physical status I (*n* = 19, 40.4%) or II (*n* = 28, 59,6%). No patients with ASA status ≥ III were included in this initial series. [Table 1] The majority (*n* = 29/47, 61.7%) of patients had undergone pelvic surgery prior to the Dexter system-assisted operation.

**Table 1:** Baseline characteristics

Number of cases			47
Age [median]			41 (IQR 36.5–48)
BMI (median) [kg/m^2^]			24.4 (IQR 21.9–28.4)
ASA classification (n/N, %)			
	1		19/47 (40.4%)
	2		28/47 (59.6%)
Previous abdominal surgery			
	no previous surgery (n/N, %)		19/47 (40.4%)
	any previous surgery (n/N, %)		28/47 (59 6%)
		caesarean section	9/47 (19.1%)
		ovarian cyst removal	6/47 (12.8%)
		endometriosis excision	5/47 (10.6%)
		appendectomy	5/47 (10.6%)
		myomectomy	5/47 (10.6%)
		diagnostic laparoscopy	2/47 (4.3%)
		cholecystectomy	2/47 (4.3%)
		sacrospinous hysteropexy	1/47 (2.1%)

**Fig. 2:**
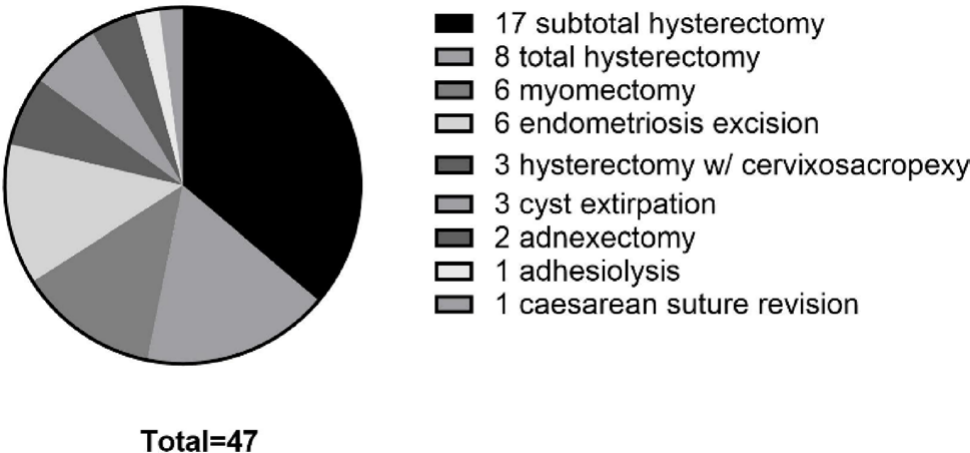
Distribution of the performed operations using the Dexter robotic system. A total of 47 cases of 9 different gynecological procedures were carried out, with subtotal and total hysterectomies constituting the majority of procedures

Nine different types of gynecological procedures were performed [Table 2; Fig. 2]. The most common procedures were benign hysterectomies (*n* = 28, 59.6%), subdivided into subtotal hysterectomy (*n* = 17, 36.2%), total hysterectomy (*n* = 8, 17.0%) and hysterectomy combined with cervicosacropexy (*n* = 3, 6.4%). Other procedures included myomectomy (*n* = 6, 12.8%), endometriosis surgery (*n* = 6, 12.8%), ovarian cystectomy (*n* = 3, 6.4%), adnexectomy (*n* = 2, 4.3%), revision of cesarean scar (*n* = 1, 2.1%) and adhesiolysis alone, without concomitant interventions (*n* = 1, 2.1%).

**Table 2:** All operations included in the study with mean operative times and standard deviation

Procedure		Number	Mean operating time [min]	Standard deviation [min]
Subtotal hysterectomy		17	117.9	17
Total hysterectomy		8	139.8	33.1
Myomectomy		6	131	42.7
Endometriosis excision		6	91.8	20.4
Hysterectomy w/ Cervicosacropexy		3	161	26
Cyst extirpation		3	88.7	30.1
Adnexectomy		2	88.5	13.4
Adhesiolysis		1	60	
Cesarean suture revision		1	129	

All 47 procedures were completed robotically without conversion to conventional laparoscopy or laparotomy. No intraoperative complications occurred. Median estimated blood loss was < 50 ml across all procedure types. No patient required intraoperative or postoperative blood transfusion. The mean duration of hospitalization (including the day of operation) for patients who underwent surgery using the Dexter robotic system was 3.3 days ± 0.85 days. Six of 47 patients (12.7%) were readmitted to the hospital. Detailed analysis of these cases revealed: One patient underwent planned completion surgery 49 days postoperatively after final pathology revealed high-grade ovarian cancer following adnexectomy for presumed benign pathology. Four patients presented as emergency readmissions (median 37.5 days, range 9–427 days) with symptoms related to underlying chronic conditions rather than surgical complications: persistent endometriosis symptoms (*n* = 2), chronic abdominal pain (*n* = 1) and menstrual complaints (*n* = 1). All four underwent transvaginal sonography demonstrating normal postoperative findings without surgical complications. Management was conservative with analgesia (*n* = 2) or observation only (*n* = 2). One patient was readmitted 28 days postoperatively for suspected cervicosacropexy mesh-related discomfort.

Mean operative times varied by procedure type and complexity. [Table 2] The longest operative time was observed for hysterectomy combined with cervicosacropexy (161.0 min ± 26.0 min), followed by total hysterectomy (139.8 min ± 33.1 min) and myomectomy (131.0 min ± 42.7 min). Subtotal hysterectomy demonstrated the shortest mean operative time among hysterectomy subtypes (117.9 min ± 17.0 min).

Pathological specimens provided context for operative complexity. For total hysterectomy, median uterine weight was 203 g (IQR 90.8–301.5 g), 206 g (IQR 115–442 g) for subtotal hysterectomy and 83 g (weights: 60 g, 83 g, 135 g) for hysterectomy combined with cervicosacropexy. For myomectomy cases, fibroid burden varied substantially with median number of 1 fibroid removed (range 1 to ≥ 3 fibroids) and median total fibroid weight of 47 g (range 6–790 g). Fibroid size was only documented in only one case (5 cm).

**Fig. 3:**
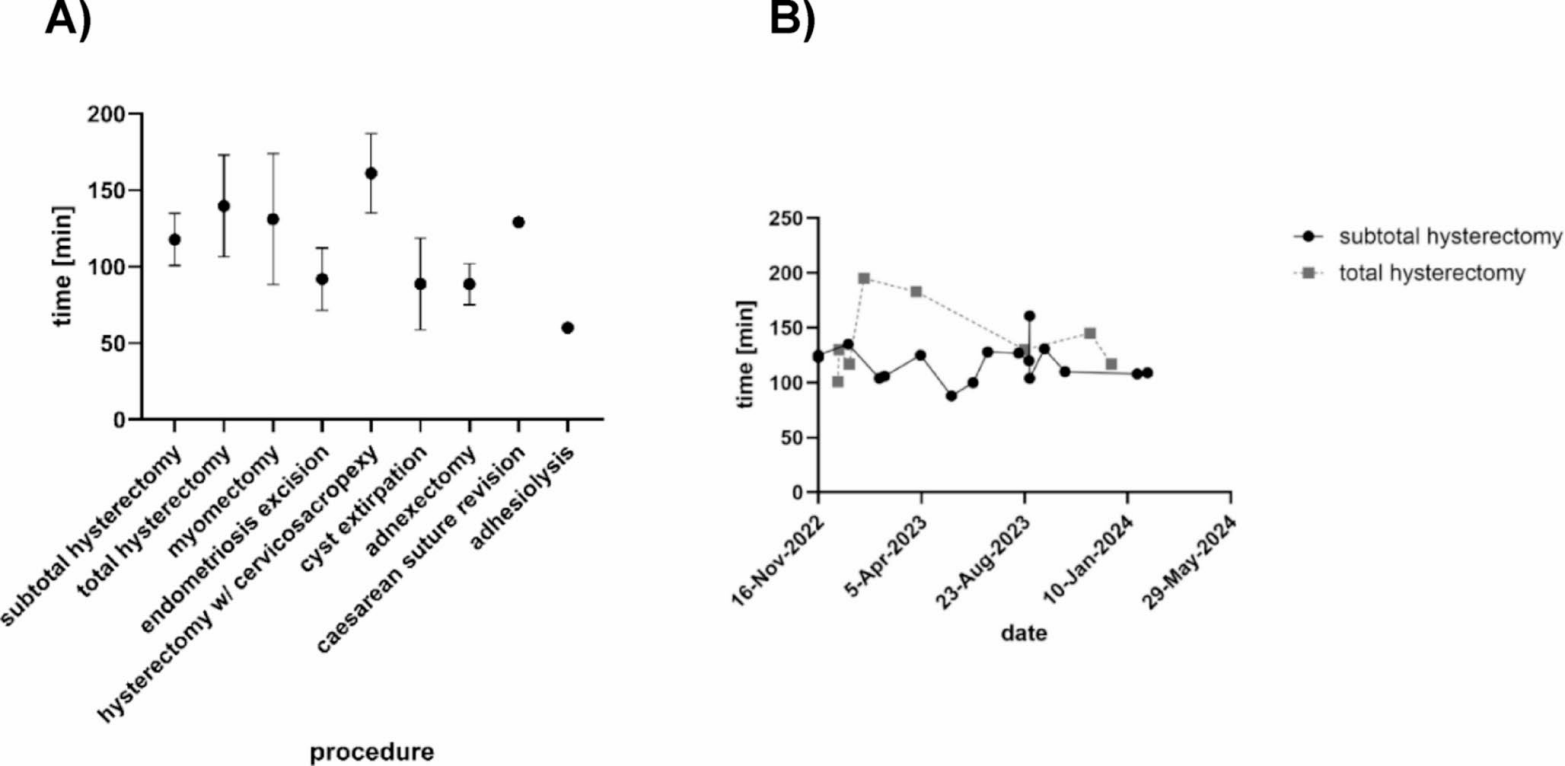
**(A)** Mean values of operation times per procedure. A discrepancy in operation times was identified between subtotal and total hysterectomy, with a mean difference of 21.9 min. **(B)** Operation times of all subtotal (*n* = 17) and total hysterectomies (*n* = 8) in the study period. No reduction of operation times was observed for either procedure

A further analysis of the duration of operation revealed differences in the duration of the various types of hysterectomy. Comparison between total and subtotal hysterectomy being the two most common procedures showed a median difference of 21.9 min (*p* = 0.11), albeit did not reach statistical significance. [Figure 3A] An analysis of the operative times for the two most prevalent interventions, namely subtotal and total hysterectomy, over the observation period revealed no substantial change or reduction. [Figure 3B].

When the variability of operative times is taken into account, we found substantial differences in the respective types of gynecological operations using the Dexter system. Despite the fact that only two cases had undergone adnexectomy (88.5 min ± 13.4 min), this procedure demonstrated the least variability in operative time. Conversely, in analyses encompassing more than five cases, myomectomy (131 min ± 42.7 min) was the procedure with the highest variability in operative time. Despite the fact that hysterectomy with cervicosacropexy (161.0 min ± 26.0 min) took the longest time, total hysterectomy (139.8 min ± 33.1 min) showed the highest variability. The operative time for subtotal hysterectomy (117.9 min ± 17.0 min) was found to be the shortest and the variability of operative times was the least.

## Discussion

This consecutive case series of 47 patients represents the first systematic evaluation of the Dexter robotic system in routine gynecological surgery. All procedures were completed without conversion to laparotomy or conventional laparoscopy and no intraoperative complications occurred. These findings demonstrate the technical feasibility of the Dexter system across a range of gynecological procedures, including hysterectomy, myomectomy, endometriosis surgery and cervicosacropexy. While previous reports have described the use of the Dexter system in urology and general surgery, data on gynecological applications have been mainly limited to hysterectomies [7, 9, 11–14]. Here the Dexter robotic system was used in a substantial proportion of cases (*n* = 47), thereby facilitating a diverse array of gynecological procedures. Our series provides foundational outcome data and operative benchmarks for centers considering adoption of this platform.

The Dexter robotic system proved to be safe for use in a wide range of gynecological procedures and a diverse patient population, irrespective of age, prior pelvic surgery, or BMI. No intraoperative complications or significant blood loss occurred during the operations, thus confirming the safety profile of the Dexter robotic system and its applicability in gynecological surgery. These findings align with reported safety outcomes of the use of other robotic surgery platform across various surgical specialties, though direct comparison would require matched control groups [15, 16]. 

The observed readmission rate of 12.7% must be contextualized. Detailed review of all six readmissions revealed that none were related to technical aspects of the Dexter platform, surgical technique or perioperative complications. Instead, readmissions reflected disease-related factors. Four cases of chronic symptom recurrence of endometriosis or dysmenorrhea, one planned reoperation for occult malignancy and one case of potential of late mesh-related discomfort. This pattern differs substantially from readmissions due to surgical site infections, bleeding or technical complications typically assessed in surgical quality metrics [17]. The rate reflects challenges of managing chronic benign gynecological conditions rather than platform-specific safety concerns.

The Dexter robotic system differs fundamentally from integrated robotic platforms through its on-demand hybrid design. The sterile console positioning allows surgeons to transition seamlessly between manual laparoscopic and robotic operation without breaking sterility – a feature not available in conventional robotic systems requiring non-sterile remote consoles. This design offers several practical advantages, including the ability for surgeons to quickly switch to manual technique for steps requiring tactile feedback and respond immediately to unexpected intraoperative findings without undocking. In our series, the hybrid capability was utilized pragmatically across for tasks such as specimen retrieval, placement of additional trocars as needed during the procedure and direct assessment of tissue quality. While we did not systematically quantify the frequency and duration of hybrid transitions in this retrospective series, this flexibility represents a key differentiating feature. Future studies should prospectively document hybrid utilization patterns to better characterize optimal use cases for this functionality.

Our data demonstrate a clear correlation between technical complexity and operative time. Consequently, the data indicate that combined procedures such as hysterectomy with cervicosacropexy (161.0 min ± 26.0 min) predictably require the longest operative time, whereas less complex procedures such as adnexectomy (88.5 min ± 13.4 min) can be performed in a shorter period of time [18]. The largest number of procedures performed with the Dexter system were hysterectomies (subtotal hysterectomy: 17; total hysterectomy: 8; hysterectomy with cervicosacropexy: 3). The collated data regarding operative times (subtotal hysterectomy M = 117.9 min; total hysterectomy M = 139.8 min; hysterectomy with cervicosacropexy M = 161 min) fall within the ranges reported in the literature for robotic-assisted hysterectomy. However, direct comparison with other platforms is limited by the absence of a control group in this study [19]. The 21.9 min difference between total and subtotal hysterectomy, although not statistically significant (*p* = 0.11), reflects the additional surgical steps required for cervical amputation and vaginal cuff closure, consistent with existing literature comparing these approaches [20, 21]. The standard deviations of operative time observed in the respective gynecological procedures provide insights into the degree of standardization across the different procedures. Subtotal hysterectomy showed the lowest standard deviation in operative time, suggesting a high degree of standardization for this procedure as shown by Nesbitt-Hawes et al. [22] The observed variations in operative times for myomectomies (SD = 42.7 min) were striking. This heterogeneity reflects inherent differences in case complexity rather than platform-specific issues. The high variability in myomectomy operative times is expected given the wide range of fibroid characteristics and varying locations, including one case with at least 3 fibroids and total weight of 790 g, compared to other cases with solitary small fibroids (6–102 g). This variability has been similarly reported for myomectomy using conventional laparoscopy and underscores the importance of stratifying cases by complexity in future comparative studies [23–25]. 

The identified differences in operative times and their variability provide an empirical foundation for the development of robotic surgery training programs. The Dexter robotic system is particularly well-suited for this purpose because of its open and modular design. The system facilitates a seamless transition between conventional laparoscopic and robotic-assisted surgical approaches during operations, thus allowing a stepwise integration of robotic-assisted surgical procedures [7, 10]. Procedures with low standard deviations, such as subtotal hysterectomy (SD = 17.0 min) or adnexectomy (SD = 13.4 min) are particularly suitable for early robotic surgery training due to their predictable operative conditions. More complex procedures with more variations such as myomectomy (SD = 42.7 min) should be integrated into advanced stages of training curricula.

We did not observe a significant reduction in operative times over the study period, likely due to evolving case selection rather than absence of a learning effect. [Figure 3B] As surgeons gained confidence with the platform, case complexity gradually increased, with more patients having prior pelvic surgery and higher BMI included in later cases. Both operating surgeons had extensive prior robotic experience, which likely facilitated rapid adaptation to the Dexter system. The manufacturer’s structured training program, including simulator exercises and proctored cases, may have contributed to the safe implementation without complications during the initial series. Future studies should employ standardized case selection and validated complexity scores to properly assess learning curves with the Dexter robotic platform.

### Limitations of the study

Several limitations should be acknowledged: This single-center retrospective case series lacks a control group, precluding direct statistical comparison with conventional laparoscopy or other robotic platforms, thereby no conclusions regarding superiority, non-inferiority or equivalence can be drawn from the dataset. The reported operative times can be contextualized against published literature. However, such comparisons are inherently limited by heterogeneity in case selection, surgeon experience, institutional workflows and patient populations across studies. The heterogenous case mix with small numbers for several procedure types limits the statistical power and generalizability of findings for less common procedures. Meaningful conclusions are primarily supported for hysterectomies (*n* = 28) and to a lesser extent, myomectomies and endometriosis surgery (*n* = 6 each). Case selection during this initial implementation phase favored lower-risk patients (ASA I-II only), limiting generalizability to higher-risk populations or complex oncological cases.

Device-specific metrics such as docking time and hybrid utilization were not systematically captured in this retrospective analysis. These metrics would be valuable for comprehensive platform evaluation and should be prospectively recorded in future studies. All procedures were performed at a high-volume academic center by surgeons with extensive prior robotic experience, which may not reflect the learning curve at centers adopting the Dexter robotic system as their first robotic platform.

Finally, the study period represents an initial experience phase where case selection and workflow evolved, potentially confounding assessment of true learning curves.

### Future perspectives

These initial findings establish a foundation for more comprehensive evaluation of the Dexter robotic system in gynecological surgery. Prospective comparative studies with matched controls, including both conventional laparoscopy and established robotic platforms, are needed to assess whether the hybrid design translates into measurable clinical advantages. Systematic quantification of hybrid utilization patterns would help determine optimal workflow integration and identify which surgical steps benefit most from robotic assistance versus manual technique.

Economic analysis comparing capital costs, maintenance requirements and per-procedure expenses against integrated robotic systems represents another important research priority, particularly for institutions evaluating platform adoption. Multi-center implementation studies would provide valuable insights into learning curves and outcomes across institutions with varying levels of robotic experience, helping establish realistic expectations for centers new to the platform.

Expansions to more complex cases, including oncologic surgery requiring lymphadenectomy, higher risk patients and technically demanding procedures, would further define the platform’s capabilities and limitations. Standardized training curricula specific to the Dexter platform should be developed and validated, as the on-demand hybrid design may offer unique advantages for stepwise skill acquisition. Long-term outcome data, including patient-reported outcomes, functional results and quality of life measures, are also needed to comprehensively assess the platform’s role in gynecological surgery.

## Conclusions

This consecutive case series demonstrates the technical feasibility of integrating the Dexter robotic system into routine gynecological practice, with successful completion of 47 procedures across nine procedure types without conversions or intraoperative complications. The on-demand hybrid design was successfully incorporated into surgical workflow, though systematic quantification of this unique feature is needed in future studies. Operative times fell within reported ranges in published literature for robotic-assisted and conventional laparoscopic gynecologic surgery and the absence of complications during the initial learning phase is encouraging. These findings provide foundational outcome data for the Dexter robotic system in gynecology and support further evaluation through prospective comparative studies to determine the platform’s advantages, optimal use cases and long-term role in minimally invasive gynecologic surgery.

## Data Availability

The European Union General Data Protection Regulation law regulates and restricts public sharing of the underlying clinical data. However, aggregated anonymized data can be disclosed upon reasonable request.
